# A Time-motion Comparison of Itemized Treatment Costs in First and Second In Vitro Fertilization Attempts: A United Kingdom Fertility Centre Experience

**DOI:** 10.36469/9855

**Published:** 2013-07-19

**Authors:** Christopher A. Jones, Olivia J. Carpinello, Dev Kumar, Louis G. Keith, Renju S. Raj, E. Scott Sills

**Affiliations:** 1 Center for Study of Multiple Birth, Chicago, Illinois USA; Vermont Center for Clinical and Translational Science, Global Health Economics Unit, University of Vermont, College of Medicine, Burlington Vermont USA; Center for Science and Society, Trinity College, University of Oxford, Oxford UK; University of Vermont College of Medicine, Department of Surgery, Burlington, Vermont USA; 2 University of Vermont College of Medicine, Department of Surgery, Burlington, Vermont USA; 3 Senior Legal Advisor, Boehringer Ingelheim, Berkshire UK; 4 Center for Study of Multiple Birth, Chicago, Illinois USA; Department of Obstetrics & Gynecology, Feinberg School of Medicine, Northwestern University, Chicago, Illinois USA; 5 Department of Obstetrics, Gynecology and Reproductive Sciences, University of Vermont College of Medicine; Burlington, Vermont USA; 6 Division of Reproductive Endocrinology, Pacific Reproductive Center, Irvine, California USA; School of Life Sciences, University of Westminster, London UK

**Keywords:** systems management, simulation laboratory, resource allocation, ivf treatments, in vitro fertilisation, cost-efficiency, time-motion analysis

## Abstract

**Objective:** To assess the difference in cost between initial and second in vitro fertilization (IVF) cycles in the United Kingdom.

**Methods:** This prospective time-motion analysis captured data on average time spent on 31 representative components of the IVF sequence as provided by clinical team members in seven categories. Audits of consumables and observations on personnel costs were made from total of 120 fertility patients undergoing initial or second IVF cycles (n=736) between 1 January 2002 and 31 December 2002 at a UK assisted fertility unit.

**Results:** Patients spent an average of 16.71±4.3 hrs with staff during an initial IVF cycle, resulting in direct personnel costs of £577.05±151.01. When consumables were included, each initial cycle cost the clinic approximately £2246.57±151.01. For second IVF cycles, patients spent significantly less time with staff compared to their first IVF cycle (6.94±2.44 hrs; p<0.05), corresponding to £257.53±90.77 in personnel cost.

**Conclusions:** This is the first economic appraisal of the IVF treatment sequence in the UK using a timemotion analysis model. Our study found that when combined with consumables, total institutional costs for second IVF cycles were significantly reduced when compared to initial cycles (£1813.12±90.77; p<0.05). Aggregating data from all IVF cycles performed within the fertility centre during the study interval, initial cycles were found to be front-loaded, resulting in £252,420 more in institutional costs as compared with subsequent IVF cycles. While these observations were registered in 2003, an inflation adjustment using recent European Commission Eurostat data for healthcare finds the difference between initial and subsequent fresh IVF cycles in present currency to be approximately £579.14 per cycle. Time-motion analysis can identify episodes of care that can be streamlined to improve outcomes and reduce cost.

## INTRODUCTION

The utilisation of advanced reproductive technologies has increased dramatically in the United Kingdom over the past 20 years. In 1992, only one in every 3,000 UK babies was conceived through in vitro fertilisation (IVF), while by 2009 the number increased to one in 50.[1] The total number of IVF patients treated, as well as the number of overall IVF cycles performed, generally trended upward during this interval. A total of 6,650 IVF cycles were performed in 1991, and (except for a brief decline in 1998-1999), this number has steadily increased each subsequent year to 37,600 by 2003. Now a decade on, these results were first reported in the doctoral dissertation by one of the authors (CAJ) and remain prescient. The number of IVF patients also increased during this time from 6,184 in 1991 to 29,300 in 2003.[2] Interestingly, the number of IVF cycles per million UK inhabitants remains lower than the rest of Europe, despite these increases[3-12] (see Figure 1). In the late 1990's, for example, only approximately 15% of the UK couples who qualified for IVF actually underwent this treatment.[13] While this low utilisation rate may be attributed to stresses associated with IVF or with the decision not to have children, these issues are not unique to UK patients. The impact of high treatment cost and the prospect of relatively low success rates could also contribute to the relatively low IVF uptake rate in the United Kingdom. In 1998 for example, only 10.8 IVF cycles per 100,000 population were funded by the NHS, which resulted in most IVF cycles being paid for by patients privately.[14]

**Figure 1.** Number of IVF Cycles per Million Inhabitants in Various European Countries from 1997-2006

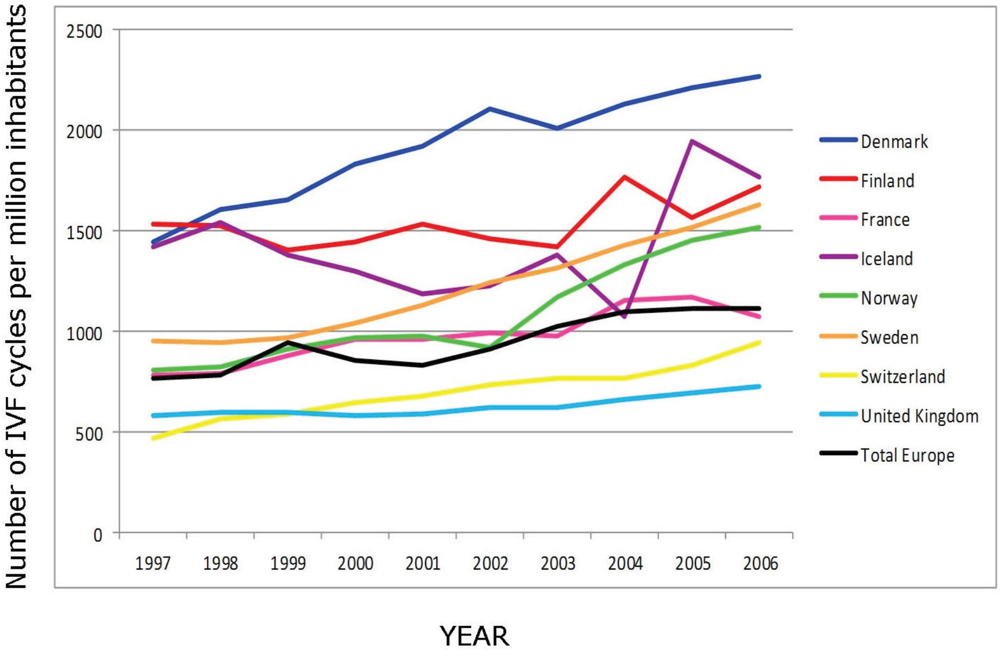

The 'Fertility Guideline for England' was among the first clinical guidelines produced from the National Institute of Clinical Excellence (NICE). This report examined cost-effectiveness of advanced reproductive technologies[15] with a focus on cost impacts of putative changes in demand for IVF in relation to the costs of treatment, as well as deliveries. Following this publication, the NHS increased its commitment to IVF funding by accepting the NICE recommendation to cover up to three full IVF cycles for patients between ages 23-39, with either a diagnosed cause of infertility or infertility of at least 3 years' duration.[16] Based on this funding proposal, an 80% increase in IVF demand in the UK population was predicted.[15] However, implementation of this guideline has not been consistently applied throughout the country. Indeed, some primary care trusts (PCTs) have placed additional criteria to determine eligibility for IVF treatment.[16]

Thus, by 2006 the increase in IVF uptake was moderate, with 44,200 IVF cycles undertaken by 34,800 patients,[2] which corresponds to an increase by 17.6% and 18.8%, respectively, from 2003. Of note, preliminary data from 2010 suggest a further increased to 57,650 cycles undertaken by 45,260 women.[17] Compared to 2003, these latter numbers represent a clinically important increase (53.5% and 54.3%, respectively).

An examination of treatment cost is useful, particularly given this observed increase in demand for IVF in the United Kingdom. The process of cost measurement encompasses a variety of methods of varying levels of complexity, the most common being based on cost accounting methods. Examples include time-motion studies (where a complex task is divided into simpler components, measured, and production and delivery times and prices computed), diary methods (in which information is self-recorded on a regular basis to help eliminate recall bias), and interviews with caregivers. Our investigation is a time-motion analysis, designed to measure differences between initial versus subsequent fresh IVF cycles regarding personnel time, consumables, and other resources and activities. Because the provision of IVF occurs against a background of general efforts to minimize overall healthcare costs by increasing efficiency, it is anticipated that time-motion analyses will become a common tool to identify opportunities to enhance care and expand access to particular medical services. Nevertheless, the current investigation is believed to be the first to apply a time-motion model specifically to IVF.

## MATERIALS AND METHODS

### Study Population and Setting

This observational analysis collected data on the average time that healthcare providers spend on specific IVF procedures, with a focus on employee costs associated with key components of the IVF process. Following registration with the Human Fertilisation & Embryology Authority (HFEA), non-identifiable and anonymous data were tabulated on patient volunteers (n=120 couples) who received fresh, non-donor gamete IVF treatment at a university-affiliated fertility centre in the United Kingdom. Our investigation included eligible IVF patients who received their treatment from 1 January 2002 to 31 December 2002. To place findings into a transferrable context, all personnel costs were reported in units of time matched to corresponding NHS reference costs. All IVF patients embarking on their first treatment at the study site were classified as an initial cycle patients, even if they had undergone previous IVF treatment elsewhere (i.e., transfers from outside clinics were designated as 'initial cycle'). Second cycle patients were designated as such only when the initial cycle was carried out at the study site. The clinic personnel at the fertility unit remained unchanged throughout. Since this analysis was confined to data on procedural and clinic-based interactive events during IVF, costs associated with particular gonadotropin regimes were outside the scope of the study.

### Components of Treatment

Patient care activities were divided into separate events classified as distinct components of treatment as follows: For initial IVF cycles, potential treatment activities were defined as follows: a) initial medical appointment; b) drug appointment; c) evening meeting; d) new patient consultation; e) baseline ultrasound scan (USS); f) scheduling of appointments; g) blood tests; h) ovarian cyst aspiration; i) follicular tracking; j) egg collection; k) intracytoplasmic sperm injection (ICSI); l) embryo transfer; m) pregnancy testing; n) counseling; o) andrology analysis; and p) referral to a GP for antenatal care. While these components were possible elements of the initial IVF cycles, not every patient underwent all of these procedures.

For subsequent (second attempt) fresh IVF cycles, components of treatment were defined as follows: a)

scheduling of appointments; b) blood tests; c) ovarian cyst aspiration; d) follicular tracking; e) egg collection; f) ICSI; g) embryo transfer; h) pregnancy confirmation; i) counseling; and j) referral to a GP for antenatal care. While these components were possible elements of a second IVF cycle, not every patient underwent all of these procedures. Subsequent cycles which utilized frozen embryos were excluded from the study.

During the initial medical appointment, patients met with a physician to discuss their diagnoses and any outstanding results or required investigations before commencing treatment. In addition, an overview of the IVF/ISCI procedure was provided. The evening meeting consisted of a presentation given by a multi-disciplinary team regarding the etiology of (and the treatment options for) infertility, as well as an opportunity for patients to ask questions. The new patient consult was uniformly supervised by senior members of the nursing team and involved signing consent forms, verifying that the necessary screening tests were current (e.g. HIV, Hepatitis B and C) and instructing the couple on how to self-administer gonadotropins. Endometrial mapping (trial embryo transfer) was also documented at this visit. Before oocyte retrieval, at least one USS was performed to monitor follicular recruitment and to minimise risk for ovarian hyperstimulation syndrome.[18] The components of egg collection included time spent by patient in the waiting room, surgical preparation, obtaining written informed consent, egg retrieval, rest and follow-up arrangements, and sperm procurement and analysis. These tasks did not differ between initial and subsequent cycles. Both a fertility doctor and an anaesthetist were present and interacted with the patient during oocyte retrieval. The components of embryo transfer included time spent by patient in the waiting room, a consultation during which written informed consent was obtained, the procedure of embryo transfer, rest and follow-up arrangements. For purposes of this study, only patients undergoing embryo transfer without ultrasound guidance were included for analysis.

Pregnancy confirmation in initial and subsequent cycles included a serum pregnancy test, diagnosis of clinical pregnancy by USS at 2-3 weeks' gestation, confirmation USS at 8 weeks to document appropriate interval growth, or care for ectopic pregnancies or pregnancies that lead to later stage miscarriage, if applicable. Multiple gestations and ectopic pregnancy were noted in the patient's record if discovered by ultrasound at any stage, but the methods of pregnancy confirmation did not differ. No selective reductions were performed during the study interval.

## Data Collection

A standard stop-watch was used to measure the duration associated with each treatment component. Physical presence of staff was recorded as the frequency of each member in attendance, and durations of staff involvement were timed and rounded to the nearest 0.08 hours (5 minutes). For each IVF treatment component, average time per task was determined by averaging the observed duration for five separate assessments of the same procedure, taken from a total of 300 observations. The distinction between which components were offered on initial versus subsequent attempts was derived from a total of 170 (34 treatment components x 5 observations) data points for initial cycles, plus 130 (26 treatment components x 5 observations) for repeat cycles. This study included a total of 125 hours (7,500 minutes) of observed activities, as carried out by 42 clinical and administrative staff.

For each staff member and each treatment component, the total time was estimated by multiplying the duration of attendance by a) the percentage of patients undergoing each treatment component and b) the frequency of attendance by each staff member. Activities were then totaled and averaged to yield the approximate per cycle time that staff devoted to initial and subsequent cycles. Observations were then multiplied by the published per-hour NHS reference costs (2003) for each staff member in 2003 British pounds sterling.[19] The result of subtracting subsequent cycles from initial cycles yielded the difference in time and cost between them.

Personnel were placed into seven categories as determined by clinical or administrative roles: a) physician/ specialist registrar; b) nurse; c) embryologist; d) andrologist; e) receptionist; f) counsellor, and g) consultant anaesthetist. Hourly personnel costs (adapted from NHS) are summarized in Table 1, as reported by the Personal Social Services Research Unit (PSSRU), University of Kent, in Unit Costs of Health and Social Care.[19] Where published UK wage estimates were unavailable, hourly salaries were calculated from a compensation audit performed within the fertility centre (McVeigh, personal communication, 2002-2005).

The 2003 costs per hour of patient contact with specialist registrars, senior house officers, practice nurses, and fertility counsellors were: £40, £35, £31, and £32, respectively (see Table 1). Estimates included all annual wages, salary overheads, board certification, ongoing training (funded by the state), and shared capital overheads. It should be noted that cost figures for fertility counsellors excluded a training component, because this may vary widely between practices (an adjustment of 25% is included in the sensitivity analysis, to account for this variation). According to the unit's 2003 data, the hourly costs for the embryological technician, receptionist and andrologist were equal, and these costs included annual wages, salary overheads, overheads with travel, and ongoing training, at £25 per hour (see Table 1). This figure did not include shared capital overheads such as joint office costs and performance-based wage variances. Accordingly, a variation of ±25% of hourly rate was included in the sensitivity analysis. Hourly costs for the consultant anaesthetists included all annual wages, salary overheads, board certification, ongoing training, and shared capital overheads in the estimation of £109 per hour of patient contact. For this component, assessment included both direct patient-related activities and essential administrative work including charting and consents (McVeigh, personal communication, 2002-2005).

**Table 1.** Summary of Various Fertility Unit Personnel Costs (in GBP/hour) for Time-motion Comparison between First and Second IVF Cycles

| Team member | £/hr |
|---|---|
| Medical Doctor (SpR) | 40.00[a] |
| Medical Doctor (SHO) | 35.00[a] |
| Medical Doctor (Consultant) | 109.00[a] |
| Nurse | 31.00[b] |
| Embryological Technician | 25.00[c] |
| Andrologist | 25.00[c] |
| Receptionist | 25.00[c] |
| Counsellor | 32.00[b] |
| Consultant Anaesthetist | 109.00[b] |

Notes: [a]Medical doctor personnel cost difference by grade, from PSSRU (2003); [b]PSSRU, 2003 (includes 25% variance in sensitivity analysis); [c]From audit of financial records at Oxford Fertility Unit, 2003 (includes 25% variance in sensitivity analysis).

### Analysis

A total of 31 elements (consumables and equipment) were included in our analysis along with capital overheads such as office rent, utilities, maintenance, and equipment services (see Table 2). This approach was employed recognising that these elements do not change appreciably between initial and subsequent treatment cycles. A per treatment cycle cost was generated for these combined services by dividing their annual cost by the total annual number of 736 treatment cycles performed during calendar year 2002 (and reported to HFEA in 2003) by the study site. Since it was assumed that patients would receive their subsequent cycles in the same calendar year as the initial treatment cycle, inflation and depreciation were excluded from analysis. This assumption was considered valid considering that >95% of patients at the study site underwent a second fresh cycle within 12 months of the initial fresh cycle. Productivity and performance of staff were regarded as stable between initial and subsequent treatment cycles (e.g., uniform personnel), so these elements were not included for analysis.

A multi-way sensitivity analysis (accomplished by varying more than one variable concurrently to examine the relationship of different parameters changing simultaneously) was performed to test the robustness of the conclusions to changes in key components by assigning high and low ranges to personnel costs and re-estimating the results accordingly. The average of these ranges was termed the 'nominal scenario' or the 'base case'. For nominal (mean), high, and low cost scenarios, summary time and cost data were analyzed in Microsoft Excel Version 2003. This analysis included staffing component costs, initial IVF cycles, subsequent fresh IVF cycles and differences between initial and subsequent cycles. The nominal scenario was used to arrive at the reported conclusions. Results were analyzed for time and financial expenditure to calculate cost differences between initial and subsequent fresh cycle attempts. An estimation of the total additional cost to the fertility centre of initial compared to subsequent fresh cycles was made using the annual number of cycles provided by the centre during the 2003 calendar year.

## RESULTS

During the 12-month study interval, a total of 120 patients underwent initial or subsequent fresh IVF cycles. The results are based on five observations for each of the 34 treatment components that make up initial cycles and five observations for each of the 26 treatment components that make up repeat fresh cycles.

### Rent and Services

The building from which the fertility centre operated was owned by the Oxford Radcliffe Hospitals NHS Trust. The cost per cycle of NHS rent and services was calculated by dividing the total cost of £224,720 for NHS rent and services by the 736 treatment cycles reported to the HFEA by the fertility centre in 2003. The costs of maintenance and other services were calculated by dividing the total cost of £517,096 by this number of treatment cycles. These calculations yielded respective per-cycle costs of £305.33 and £702.58 for NHS rent, services and other costs (see Table 2). These per cycle estimations of consumables and overheads were constant between initial and subsequent treatment cycles. Therefore, the incremental difference calculations between initial and subsequent cycles were not affected by these running costs.

**Table 2.** Outline of Fixed Expense and Consumable Cost Data for Time-motion Comparison between First and Second IVF Cycles in a UK Fertility Unit (GBP, 2003)

| Consumables Tracked | £ |
|---|---|
| Anesthesia supplies | 150.00 |
| HFEA license fee | 103.00 |
| Ovarian cyst aspiration consumables | 50.00 |
| Medications provided by unit | 35.00 |
| Laboratory Scanner | 33.97 |
| ICSI microscope | 30.00 |
| Baseline Ultrasound scan | 25.00 |
| Culture media | 25.00 |
| Sperm procurement: Culture media | 25.00 |
| Initial pregnancy USS | 25.00 |
| Follow-up pregnancy USS | 25.00 |
| Cryobiology reagents | 24.00 |
| Embryo transfer: Laboratory equipment | 20.38 |
| Andrology assay consumables | 20.00 |
| Sperm procurement: Assay consumables | 15.00 |
| Sperm procurement: Plastic ware | 15.00 |
| Embryo transfer: Plastic ware | 15.00 |
| Embryo transfer: Assay consumables | 15.00 |
| Assay reagents & other consumables | 15.00 |
| ICSI plastic ware | 15.00 |
| ICSI consumables | 15.00 |
| Single-use plastic ware, miscellaneous | 15.00 |
| Miscellaneous furniture (3 pieces) | 14.27 |
| Pregnancy confirmation assay | 10.00 |
| Phlebotomy consumables | 10.00 |
| Pregnancy diagnosis miscellaneous disposables | 7.00 |
| Ectopic/miscarriage surgical management (diagnostic supplies and consumables) | 1000.00 |
| **IVF Unit Fixed Expenses** | |
| NHS lease & services | 305.33 |
| Other physical plant overhead | 702.58 |

ICSI=intra-cytoplasmic sperm injection; USS=ultrasound scan; HFEA=Human Fertilisation and Embryology Authority; NHS=(UK) National Health Service

## Actual Utilization of Components of Care

Although each IVF patient could have required all listed components of treatment, this did not occur in each initial or subsequent fresh treatment cycles for a number of reasons. The most important factor affecting utilisation of particular treatment components included patient non-compliance and medical non-necessity. For initial cycles, the evening meeting was attended by approximately 60% of patients, ovarian cyst aspiration (pre-IVF) was necessary in only 5% of patients, confirmatory ultrasound scans at 2 and 8 weeks were required for 25% of patients, and additional investigations for ectopic pregnancies were required for 5% of patients. Additionally, counseling was attended by 10% of patients, 40% were deemed candidates for ICSI, and a referral to a GP for antenatal care occurred in 40% of cases. Counseling, ICSI and GP referral requirements were the same for initial and subsequent treatment cycles.

## Time and Cost Comparison: Initial vs. Second IVF Cycle

Table 3 demonstrates that nurses spent the most time with patients (7.34±1.77 hrs), whereas counsellors spent the least (0.05±0.02 hrs) in initial IVF cycles. The specialist registrar and embryological technician also spent a substantial amount of time (2.56±0.51 and 3.06±0.93 hrs, respectively) in direct patient contact during initial cycles. The most expensive components of treatment for the specialist registrar were at cycle start, and included the initial medical appointment (£25±5), the evening meeting (£24±2.40), the new patient consultation (£20±2) and baseline ultrasound (£10±2). For the embryologist, the cost per cycle largely came from the evening meeting (£15±2.55), egg collection (£43.75), follow-up procedures during the period of egg collection (£25±6.25) and ICSI (£10±1.70). In total, patients spent an average of 16.71±4.3 hours with staff during an initial IVF cycle. This staffing time represented £577.05±151.01 (2003) per initial fresh IVF cycle. When consumables were included, each initial cycle cost the IVF clinic approximately £2246.57±151.01 (Table 3).

**Table 3.** Average Personnel Requirements and Costs Associated with First and Second fresh IVF Cycles in a UK Fertility Unit, as Used for Time-motion Analysis

| Initial IVF cycle | Time (hrs) | | Expenditure (£) | |
|---|---|---|---|---|
| | Nominal | Weighted uncertainty | Nominal | Weighted uncertainty |
| SpR | 2.56 | 0.51 | 102.59 | 20.31 |
| Fertility nurse | 7.34 | 1.77 | 227.61 | 54.87 |
| Embryological technician | 3.06 | 0.93 | 76.56 | 23.31 |
| Andrologist | 1.17 | 0.25 | 29.25 | 6.25 |
| Receptionist | 1.61 | 0.49 | 40.25 | 9.75 |
| Counsellor | 0.05 | 0.02 | 1.60 | 0.54 |
| Consultant anaesthetist | 0.91 | 0.33 | 99.19 | 35.97 |
| Aggregate time for all staff | 16.71a | 4.3 | | |
| Aggregate personnel cost for all staff | | | 577.05 | 151.01 |
| Total cost (personnel + consumables) | | | 2246.57[a] | 151.01 |
| **Second IVF cycle** | **Time (hrs)** | | **Expenditure (£)** | |
| | **Nominal** | **Weighted uncertainty** | **Nominal** | **Weighted uncertainty** |
| SpR | 0.49 | 0.19 | 19.79 | 7.57 |
| Fertility nurse | 3.17 | 1.02 | 98.18 | 31.60 |
| Embryological Technician | 2.03 | 0.68 | 50.63 | 17.08 |
| Andrologist | 0.13 | 0.06 | 3.19 | 1.50 |
| Receptionist | 0.39 | 0.22 | 9.75 | 5.50 |
| Counsellor | 0.05 | 0.02 | 1.60 | 0.54 |
| Consultant Anaesthetist | 0.68 | 0.25 | 74.39 | 26.98 |
| Aggregate time for all staff | 6.94[a] | 2.44 | | |
| Aggregate personnel cost for all staff | | | 257.53 | 90.77 |
| Total cost (personnel + consumables) | | | 1813.12[a] | 90.77 |

Notes: SpR=specialist registrar; all costs reported as British Pounds Sterling (2003); [a]comparison of matched parameters between first and second IVF cycles, p<0.05 (by Student's t-test)

During subsequent fresh IVF cycles, nurses again spent the most time with patients (3.17±1.02 hrs), in contrast to counsellors who spent the least amount of time with patients (0.05±0.02 hrs). The embryological technician spent a significant amount of time with patients (2.03±0.68 hrs), although the specialist registrar spent much less time during subsequent cycles (0.49±0.19 hrs). In fact, the consultant anaesthetist spent more time with patients during subsequent fresh IVF cycles (0.68 ±0.25 hrs) than did the specialist registrar. The total amount of time patients spent with staff during subsequent fresh IVF cycles was much less than with initial cycles, at 6.94±2.44 hours. This time equated to £257.53±90.77 (2003), whereas each subsequent fresh cycle cost the clinic approximately £1813.12±90.77 including consumables (see Table 3).

This analysis shows that 9.77±4.94 more staffing hours were spent with patients during initial IVF cycles compared to subsequent fresh IVF cycles. This decrease in staffing time for repeat cycles yielded a £319.52±176.19 difference between initial and subsequent fresh cycles, and the diminished use of consumables on subsequent treatment cycles accounted for an average cost-reduction of £113.93. Accordingly, subsequent fresh IVF cycles cost, on average, £433.45±176.19 less than initial cycles. Costs were applied in 2003 prices and all consumables were included in the analysis. A multi-way sensitivity analysis incorporating best case/worst case assumptions of hourly staffing costs demonstrated that this economic difference in total cost ranges from £402.48±162.00 with low hourly staffing cost assumptions, up to £596.91±224.57) with premium hourly staffing cost assumptions.

### Annual Difference

These data show that subsequent fresh IVF cycles cost £433.45 less than initial cycles, with the majority of the cost difference attributable to personnel hours. For some IVF patients, the actual individual cost differential is marginal when compared to other aspects of clinical treatment. From the perspective of the fertility unit, however, this small per cycle difference becomes important particularly as the number of IVF cycles performed enlarges. Considering the 790 IVF cycles performed within the fertility centre during 2003, the initial cycles were found to cost £252,420 more than subsequent cycles.

## DISCUSSION

Although our data are based on observations made in 2003, after adjusting for inflation using the European Commission Eurostat data for health care through July of 2012, the current difference between subsequent and initial fresh cycles may be calculated at £579.14 per cycle.[20] When the cost of time-intensive staff (i.e., doctors, embryologists, andrologists, and nurses) is high, this cost gap between initial and subsequent cycle attempts will necessarily be high, as well. Confirmation that subsequent fresh IVF cycles cost less than initial IVF cycles is not entirely unexpected, although precisely quantifying this gap has not been previously reported. Indeed, the detailed cost analysis presented here can offer insights to optimise IVF clinic management with a view to improve care quality. Specifically, our research provides ample evidence that the IVF nursing staff provide proportionally more time to couples in a given IVF cycle than other healthcare personnel. Whether this observation may be generalised to other IVF clinics in the UK or elsewhere is speculative. Comparing specific components of treatment, the largest burden on staff during an IVF cycle occurred during the new patient consultation and was provided by not only the specialist registrar and embryologist, but also by the fertility nurse.

To our knowledge, this is the first published analysis of its kind to examine the components of treatment, personnel hours, and differences between initial and subsequent fresh cycles related to IVF. Additional studies will be helpful to identify institutional variation regarding the distribution of hours spent by each type of personnel, since particular IVF units may allocate responsibilities differently. We anticipate that future analyses will validate a reproducible difference in cost between initial and subsequent fresh IVF cycles, due to the large investment of personnel time during initial IVF cycles which is absent in the repeat attempts for IVF.

Because an 'IVF industry' appears to grow in parallel with desires to reduce the overall cost of healthcare, it is possible that time-motion analysis will be a model more widely deployed to improve outcomes and reduce costs for fertility patients. By identifying the most time-intensive and personnel-intensive components of the IVF process, this study may assist IVF clinics in identifying cost-saving measures, whether by reducing particular personnel hours or by workload redistribution. Additionally, by recognizing that initial compared to repeat IVF cycles cost the clinic significantly more, certain policies may be adopted that promote continuity of care, reduced attrition, and cost-savings for both clinic and the couples as they undergo repeat cycles. The magnitude of a bimodal cycle cost as observed here requires further testing in a clinical trial environment, but is likely to underscore the importance of continuity of care. This ensures that the record as well as the corporate memory of patient care is available at every patient visit. Without a continuity of care program in place, it is impossible to determine whether care is medically or cost-effective because there may be insufficient follow-up. All too often, disjointed care leads to poor communication, needless duplication in laboratory testing, and increased health care spend. By streamlining the pathway from initial consultation through to completion of the final cycle, a pricing system can be developed that avoids repetition and waste of healthcare resources.

It remains a matter of debate whether the price of initial or subsequent IVF cycles should be adjusted. Some clinics maintain a financial plan whereby patients can pay a higher fee for the initial IVF treatment in order to receive a discount on subsequent treatments.[21] Other plans promote included services such as three 'free' counselling sessions when self-paying patients pay a flat fee for IVF.[22] In other centers, patients are offered a substantial rebate if they do not deliver a baby after 3 cycle attempts. An important characteristic of these outcome-based or 'shared risk' plans is that they deter patients from switching providers or dropping out without a rational reason for doing so. Not only does such a plan encourage the desired continuity of care and minimization of unnecessary repeated expenditures on subsequent treatment, but it also implicitly acknowledges the economic differences between initial and subsequent IVF cycles.

Despite the fact that the base data used for the analysis are now nearly a decade old, we anticipate the findings to continue to be economically useful because of the standardized components of fresh IVF cycles. We believe this analysis is applicable to other settings, particularly in the United Kingdom, Australia, United States and other regions where a shared-decision making approach to IVF prevails. The experience within this fertility centre is perhaps unique in the level of transparency offered with regards to specific cost components of IVF treatment available in the United Kingdom.

Nevertheless, our investigation is not without its limitations. Foremost among these is the fact that only fresh cycles were observed during this limited time period. Success rates of frozen embryo (FET) cycles have been increasing over the past decade and represent a growing proportion of IVF clinic activity.[2] Given the reduced monitoring associated with FET cycles, we believe inclusion of such cycles would offer additional support to our conclusions that subsequent cycles are less expensive than initial IVF treatment. At the time of this study, it was estimated that 25% of patients in the centre elected to use frozen embryos (McVeigh, personal communication, 2002-2005). However, it is unknown how many patients decline to use frozen embryos in favour of undertaking a second fresh IVF cycle. Insurance coverage undoubtedly plays a large role in this decision, as some jurisdictions mandate no additional fresh IVF cycles until the patient's supply of frozen embryos is exhausted, whereas others do not cover FET cycles at all. An additional weakness is the fact that observations were performed on a limited sample size, over a limited duration of time, with only five observations made for each component of care during initial and subsequent fresh IVF cycles. However, no prior team has conducted a similar time-motion analysis on an IVF patient sample of any size, to our knowledge. While our assessment of IVF treatment costs would have been strengthened by including costs associated with medications required to complete this treatment,[23] this represents the focus of additional research which we hope to present later.

## CONCLUSIONS

Initial IVF cycles require more staffing time and resource allocation than subsequent attempts. The additional cost of initial cycles is largely due to early-stage introductory sessions that are staff and resource-intensive. Since introductory sessions are cost-intensive, clinics might improve cost-efficiency by offering video or electronic materials for the introductory stages, rather than allocating expensive personnel to conduct these components of treatment. However, this cost-saving change would occur at the expense of personal contact by which the couple's psychological needs are treated in parallel with the patient's physical needs. Alternative models include outcome-based pricing, in which larger initial investments are made by the patient with promised savings during subsequent cycles. Such models promote continuity of care, reduce unnecessary repeat expenditures, and address the financial discrepancies between initial and subsequent IVF cycles. We contend that the greatest satisfaction for both the patient and clinic should be achieved when fertility services maintain individualized care of patients but are designed to account for the greater consumption of resources and higher financial costs of initial IVF cycles. We agree that overall cost of IVF is an important medical issue which can limit access to care for many individuals in need. Hopefully, the methodology described here can be used by other IVF providers to enhance institutional efficiency and reduce overall costs, thereby resulting in net savings which can be passed on to our patients.

## ACKNOWLEDGEMENTS

The authors are grateful to Enda McVeigh, MD (Oxford Fertility Unit, UK) and Paul Chamberlain, MD (John Radcliffe Hospital, UK) for valuable guidance and direction during this research.

### Funding

This investigation received support from the British Overseas Research Award, the Oxford Bursary Award, and the American Friends of Christ Church (Oxford) Award.

### Conflict of Interest Declaration

The authors declare they have no competing interests.

## References

[1] HFEA: Human Fertilisation and Embryology Authority, 2011. Fertility treatment in 2010: trends and figures. http://www.hfea.gov.uk/docs/2011-11-16_-_Annual_Register_Figures_Report_final.pdf. Accessed September 20, 2012.

[2] HFEA: Human Fertilisation and Embryology Authority, 2008. A long term analysis of the HFEA Register data (1991-2006). http://www.hfea.gov.uk/docs/Latest_long_term_data_analysis_report_91-06.pdf. Accessed June 19, 2012.

[3] ESHRE: The European IVF-monitoring programme, for the European Society of Human Reproduction and Embryology. Assisted reproductive technology in Europe, 1997: Results Generated from European Registers by ESHRE. *Hum Reprod* 2001;16:384-91.

[4] ESHRE: The European IVF-monitoring programme, for the European Society of Human Reproduction and Embryology, Nygren KG, Nyboe Andersen A. Assisted Reproductive Technology in Europe, 1998: Results Generated from European Registers by ESHRE. *Hum Reprod* 2001;16:2459-71.

[5] ESHRE: The European IVF monitoring programme. Assisted Reproductive Technology in Europe, 2002. Results Generated from European Registers by ESHRE. *Hum Reprod* 2002;21:1680-97.

[6] ESHRE: The European IVF-monitoring programme, for the European Society of Human Reproduction and Embryology, Nyboe Andersen A, Gianaroli L, Nygren KG. Assisted Reproductive Technology in Europe, 2000. Results Generated from European Registers by ESHRE. *Hum Reprod* 2004;19:490-503.

[7] ESHRE: The European IVF-monitoring programme, for the European Society of Human Reproduction, and Embryology. Assisted reproductive technology in Europe, 2001: Results generated from European Registers by ESHRE. *Hum Reprod* 2005;20:1158-76.

[8] Andersen AN, Goossens V, Ferraretti AP *et al.* Assisted reproductive technology in Europe, 2004: Results generated from European registers by ESHRE. European IVF-monitoring (EIM) Consortium; European Society of Human Reproduction and Embryology (ESHRE). *Hum Reprod* 2008;23(4):756-71.

[9] Andersen AN, Goossens V, Gianaroli L, *et al.* Assisted reproductive technology in Europe, 2003: Results generated from European registers by ESHRE. *Hum Reprod* 2007;22:1513-25.

[10] Nyboe Andersen A, Goossens V, Ferraretti AP, *et al.* Assisted reproductive technology in Europe, 2004: Results generated from European registers by ESHRE. *Hum Reprod* 2008;23:756-71.

[11] Nyboe Andersen A, Goossens V, Bhattacharya S, *et al.* The European IVF-monitoring Consortium for the European Society of Human Reproduction and Embryology. Assisted reproductive technology and intrauterine inseminations in Europe, 2005: Results generated from European registers by ESHRE. *Hum Reprod* 2009;24:1267-87.

[12] de Mouzon J, Goossens V, Bhattacharya S, *et al.* The European IVF-monitoring Consortium, for the European Society of Human Reproduction and Embryology. Assisted Reproductive Technology in Europe, 2006: Results Generated from European Registers by ESHRE. *Hum Reprod* 2010;25:1851-62.

[13] Collins J: An international survey of the health economics of IVF and ICSI. *Hum Reprod Update* 2002;8:265-77.

[14] Devlin N, Parkin D: Funding fertility: Issues in the allocation and distribution of resources to assisted reproduction technologies. *Hum Fertil (Camb)* 2003;6:S2-6.

[15] NICE: National Institute of Clinical Excellence, 2004. Costing clinical guidelines: Fertility (England). SECTA Partners for change. http://www.nice.org.uk/nicemedia/live/10936/39776/39776.pdf. Accessed July 28, 2012.

16 NHS: UK National Health Service, 2011. Choices: Can I get IVF treatment on the NHS? http://www.nhs.uk/chq/Pages/889.aspx?CategoryID=54&SubCategoryID=127. Accessed June 19, 2012.

17 HFEA: Human Fertilisation and Embryology Authority, 2012. Latest UK IVF Figures: 2009 and 2010. http://www.hfea.gov.uk/ivf-figures-2006.html. Accessed June 19, 2012.

18 Alper MM, Smith LP, Sills ES: Ovarian hyperstimulation syndrome: current views on pathophysiology, risk factors, prevention, and management. *J Exp Clin Assist Reprod* 2009;6:3.

19 Netten A, Curtis L. Unit costs of health and social care 2003, Personal Social Services Research Unit (PSSRU). University of Kent, Canterbury: PSSRU. http://www.pssru.ac.uk/pdf/uc2003/uc2003.pdf. Accessed July 28, 2012.

20 Eurostat: Measuring inflation: The Harmonised Index of Consumer Prices (HICP) - Annual percentage changes, 2012. http://epp.eurostat.ec.europa.eu/inflation_dashboard/. Accessed September 5, 2012.

21 Bristol Centre for Reproductive Medicine: Self funding costs, 2005. www.repromed.co.uk Accessed March 1, 2005.

22 Bristol Centre for Reproductive Medicine: Self funding costs, 2012. www.repromed.co.uk Accessed June 19, 2012.

23 Bouwmans CA, Lintsen BM, Eijkemans MJ, *et al.* A detailed cost analysis of in vitro fertilization and intracytoplasmic sperm injection treatment. *Fertil Steril* 2008;89(2):331-41.
